# Stimulation of Neurite Outgrowth in Cerebrocortical Neurons by Sodium Channel Activator Brevetoxin-2 Requires Both N-Methyl-D-aspartate Receptor 2B (GluN2B) and p21 Protein (Cdc42/Rac)-Activated Kinase 1 (PAK1)

**DOI:** 10.3390/md20090559

**Published:** 2022-08-31

**Authors:** Suneet Mehrotra, Marsha L. Pierce, Shashank M. Dravid, Thomas F. Murray

**Affiliations:** 1 Department of Pharmacology and Neuroscience, School of Medicine, Creighton University, Omaha, NE 68178, USA; 2 Omeros, Seattle, WA 98119, USA; 3 Department of Pharmacology, College of Graduate Studies, Midwestern University, Downers Grove, IL 60515, USA

**Keywords:** brevetoxin 2 (PbTx-2), voltage-gated sodium channel (VGSC), N-methyl-D-aspartate (NMDA) receptor 2b (GluN2B), voltage-gated calcium channel (VGCC), p21 protein (Cdc42/Rac1)-activated kinase 1 (PAK1), L-type calcium channel, sodium–calcium exchanger, neurite outgrowth, dendritic arborization, miniature excitatory post-synatpic currents (mEPSCs)

## Abstract

N-methyl-D-aspartate (NMDA) receptors play a critical role in activity-dependent dendritic arborization, spinogenesis, and synapse formation by stimulating calcium-dependent signaling pathways. Previously, we have shown that brevetoxin 2 (PbTx-2), a voltage-gated sodium channel (VGSC) activator, produces a concentration-dependent increase in intracellular sodium [Na^+^]_I_ and increases NMDA receptor (NMDAR) open probabilities and NMDA-induced calcium (Ca^2+^) influxes. The objective of this study is to elucidate the downstream signaling mechanisms by which the sodium channel activator PbTx-2 influences neuronal morphology in murine cerebrocortical neurons. PbTx-2 and NMDA triggered distinct Ca^2+^-influx pathways, both of which involved the NMDA receptor 2B (GluN2B). PbTx-2-induced neurite outgrowth in day in vitro 1 (DIV-1) neurons required the small Rho GTPase Rac1 and was inhibited by both a PAK1 inhibitor and a PAK1 siRNA. PbTx-2 exposure increased the phosphorylation of PAK1 at Thr-212. At DIV-5, PbTx-2 induced increases in dendritic protrusion density, p-cofilin levels, and F-actin throughout the dendritic arbor and soma. Moreover, PbTx-2 increased miniature excitatory post-synaptic currents (mEPSCs). These data suggest that the stimulation of neurite outgrowth, spinogenesis, and synapse formation produced by PbTx-2 are mediated by GluN2B and PAK1 signaling.

## 1. Introduction

Brevetoxin 2 (PbTx-2) is a member of a family (brevetoxins 1–10) of potent lipid-soluble neurotoxins produced by the marine dinoflagellate *Karena brevis* [1]. PbTx-2 is a voltage-gated sodium channel (VGSC) activator (gating modifier) that increases intracellular sodium [2] and augments N-methyl-D-aspartate (NMDA) receptor (NMDAR) signaling [3], resulting in an increase in intracellular calcium [4,5] and the promotion of neurite outgrowth in immature cerebrocortical neurons [6]. Notably, PbTx-2 administered following a stroke produced increased neurite outgrowth and spinogenesis in a murine photothrombotic stroke model that corresponded with improved motor function [7]. 

Activity-dependent control of neuronal development involves calcium and neurotrophic signaling. Calcium (Ca^2+^) signaling involves Ca^2+^ influx pathways, including NMDARs and voltage-gated Ca^2+^ channels (VGCCs) [8,9,10]. NMDARs belong to the family of ionotropic glutamate receptors and play a critical role in activity-dependent development and plasticity [8], dendritic arborization [11,12], spine morphogenesis [13], and synapse formation [14] by stimulating Ca^2+^-dependent signaling pathways [14]. The NMDAR pathway contributes to neuronal migration during brain development [15]. NMDA receptors stabilize synapses through a feedback mechanism that alters the NMDA receptor subunit composition [16].

Most NMDARs in the mammalian CNS are comprised of 2 GluN1 and 2 GluN2 subunits. NMDA receptors require both the co-agonist glycine and glutamate binding for activation, with GluN1 subunits providing the glycine binding site and GluN2 providing the glutamate binding site [17]. There are four subtypes of GluN2 subunits (GluN2A-D) that confer distinct biophysical and pharmacological properties, which may affect their ability to interact with different intracellular signaling molecules [17,18,19]. During early cortical development, NMDARs and other amino acid-activated receptors (such as GABA receptors) are found on neuroblasts prior to their development into functioning neurons [20]. The GluN2B subunit is expressed early in the postnatal period and decreases as the animal matures, while the GluN2A subunit increases to its highest levels in the adult [21]. The GluN2C subunit continues to increase in neurons from early postnatal detection through adulthood in rat cerebellar granule cells [22]. The GluN2D subunit is expressed in Purkinje cells during the first 8 days of postnatal life [23]. Thus, the general pattern of NMDAR-subunit expression appears as the ubiquitous GluN1 subunit throughout development and adulthood and as high levels of GluN2B and GluN2D early in development, which decrease thereafter, whereas GluN2A and GluN2C subunit expression increase into adulthood. The identity of the GluN2 subunit determines many of the biophysical and pharmacological properties of NMDARs and may also influence NMDAR assembly, downstream signaling, receptor trafficking, and synaptic targeting [18]. The GluN2A subunit confers a lower affinity for glutamate, distinctly faster kinetics, greater channel open probability, and more prominent Ca^2+^-dependent desensitization than the GluN2B subunit, which confers slower channel kinetics and reduced open probability. The GluN2C and GluN2D subunits are characterized by low conductance openings and reduced sensitivity to Mg^2+^ block [18].

Activity-dependent changes have been observed in the synaptic density of GluN2A and GluN2B [24,25]. Synaptic and extra-synaptic NMDA receptors have distinct subunit compositions and functional roles [26,27,28,29], leading to unique physiological roles for each GluN2 subunit. Chronic neuronal activity promotes the ubiquitylation (Ub) and proteasomal degradation of the GluN1 and GluN2B subunits, shifting the balance from mostly GluN2B-containing to predominantly GluN2A-containing complexes [24]. The loss of GluN2B is sufficient to cause impairment of long-term depression (LTD), resulting in reduced spine density and learning deficits [30]. Regardless of subunit composition, synaptic NMDARs activate cellular antioxidant defenses [31], initiate signaling through extracellular signal-related kinases (ERKs) [32], affect histone acetylation [33], suppress death pathways [34], and activate cAMP response element binding proteins (CREBs) [32], a transcription factor that regulates gene expression, important for survival and plasticity. In contrast, extra-synaptic NMDARs inhibit cell survival pathways [35], induce mitochondrial dysfunction, and activate pro-death molecules [36].

The NMDAR regulates neuronal intracellular Ca^2+^, which is detected by the Ca^2+^ sensor calmodulin (CaM). This Ca^2+^ binds to CaM, which in turn activates the multifunctional CaM kinase (CaMK) family of serine/threonine kinases, including CaMKI, CaMKII, and CaMKIV [37]. Among these, CaMKII is an abundant protein in the adult brain [38]. The activation of CaMKII following GluN receptor activation has been shown to be important for activity-dependent spine plasticity [39] and the induction of long-term potentiation [40].

The NMDAR may regulate spine morphological plasticity by modulating the activity and regulation of the actin cytoskeleton. Rho family GTPases, including RhoA, Rac1, and Cdc42, are key regulators of actin cytoskeleton dynamics and, therefore, the most likely link between NMDAR activity and spine plasticity [41,42]. RhoA blocks dendritic initiation, reducing branching and growth [43]. Rac1 is directly modulated by guanine–nucleotide-exchange factors (GEFs) that promote the formation of active GTP-bound Rac and GTPase-activating proteins (GAPs), which catalyze GTP hydrolysis, leading to increased levels of inactive GDP-bound Rac [44]. Active Rac1 leads to lamellipodia formation [45], spine formation, dendrite initiation, elongation, and branching complexity [46,47,48]. The Rho family GTPases Cdc24 and Rac1 play a critical role in the formation and organization of cortical actin networks [14].

A key effector of the Rho GTPases is P21 protein (Cdc42/Rac)-activated kinase 1 (PAK1), which has been shown to affect both spine properties and cortical synaptic function [49,50]. The PAK family is a member of Ser/Thr kinases and is classified into two distinct groups: Group A is comprised of PAKs 1–3, and Group B consists of PAKs 4–6. All Group A PAKs bind to Cdc42 and Rac1 and are strongly activated upon binding to these GTPases [51]. Group B PAKs bind to Cdc42 and, to a lesser extent, Rac1; however, unlike Group A PAKs, this binding does not lead to an activation of the kinase. Rather, group B PAK binding affects the localization of Group B kinases and not their activation [52]. The PAK1–Cdc42/Rac1 interaction and binding (CRIB) domain is located in the N-terminal region, and the C-terminal has a kinase domain [53]. Rac1/Cdc42 association with the CRIB domain relieves PAK1 auto-inhibition, resulting in the autophosphorylation and activation of the protein kinase domain, important for regulating cytoskeletal dynamics. PAK1 is also known to indirectly affect cofilin function via the activation of phosphorylation of the LIM kinase (LIMK), which, in turn, phosphorylates and inactivates cofilin [54]. This process is essential for the regulation of F-actin turnover and the formation of protrusions [55].

The objective of the present study was to elucidate the downstream signaling mechanisms by which PbTX-2 influences neuronal morphology in immature cerebrocortical neurons. In this study, we report the involvement of GluN2B but not GluN2A, –2C, or –2D NMDA receptor subtypes in PbTx-2-stimulated neurite outgrowth. Additionally, PbTx-2 affects neurite outgrowth via CaMKII and Rac but not RhoA. Moreover, PbTx-2 induced neurite outgrowth and dendritic arborization involves PAK1. Finally, VGSC activation regulates actin cytoskeleton dynamics, leading to increased dendritic complexity.

## 2. Results

### 2.1. NMDA-Induced Ca^2+^ Influx Involves GluN2B but Not GluN2A, –2C, or –2D Complexes in Cerebrocortical Neurons

**Figure 1:**
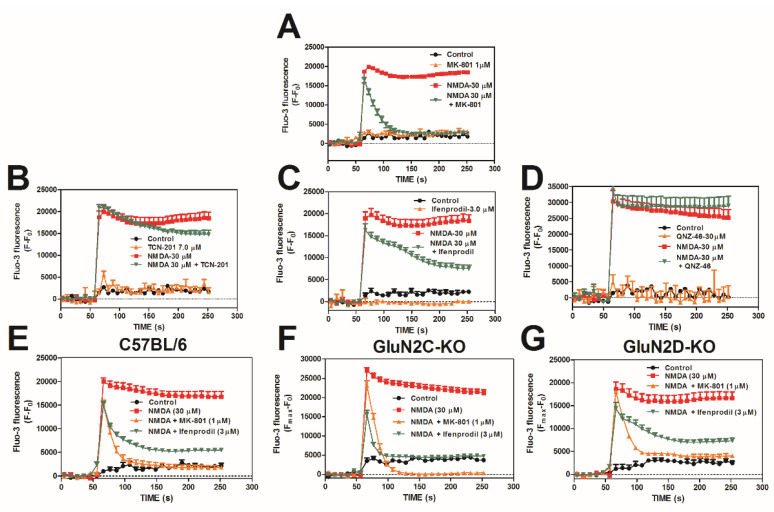
NMDA-induced Ca^2+^ influx involves GluN2B but not GluN2A, -2C, or -2D complexes in cerebrocortical neurons. Pharmacologic evidence indicating that NMDA-induced Ca^2+^ influx involves GluN2B but not GluN2A or -2C/2D complexes in cerebrocortical neurons via interrogation with NMDA channel blocker MK-801 (**A**), non-competitive GluN2A antagonist TC-201 (**B**), non-competitive GluN2B antagonist ifenprodil (**C**), and GluN2C and 2D antagonist QNZ-46 (**D**). Genetic evidence confirming that GluN2C and GluN2D NMDAR subtypes are not required for NMDA-induced Ca^2+^ influx in cerebrocortical neurons in control C57BL/6 (**E**), GluN2C-KO (**F**), and GluN2D-KO (**G**) in DIV 10–12 cerebrocortical neurons. This experiment was repeated in triplicate. Data shown represent the mean ± SEM of 3 experiments.

NMDA-induced Ca^2+^ influx was assessed in DIV 10–12 cerebrocortical neurons loaded with Fluo-3 and exposed to a 30 µM concentration of NMDA. NMDA (30 µM) produced a rapid increase in intracellular Ca^2+^ ([Ca^2+^]_i_), which was blocked by pretreatment of 1 µM MK-801 (NMDAR channel blocker, Figure 1A), resulting in a 75% reduction in the area under the curve (Figure S1A). As depicted in Figure 1, exposure of neurons to 30 µM NMDA produces a transient peak influx of Ca^2+^, followed by a plateau phase. Inasmuch as MK-801 is an open channel blocker (uncompetitive), it had little effect on the transient peak response but dramatically reduced the plateau response [56]. In order to pharmacologically determine which NMDAR subtypes are involved, we first used 3-chloro-4-fluoro-N-[(4-[(2-(phenylcarbonyl)hydrazino)carbonyl]phenyl)methyl]-benzenesulfonamide (TCN-201), a non-competitive antagonist for GluN2A (GluN2A IC_50_ = 0.109 µM, >30 µM for GluN2B or GluN2D) [57]. Glycine can surmount the inhibition by TCN-201 in functional Ca^2+^ imaging assays even though TCN-201 is minimally effective in displacing glycine site antagonists in radioligand binding assays [58]. Thus, we reduced the concentration of glycine to 1 µM in Locke’s buffer. TCN-201 (7 µM) did not inhibit NMDA-induced Ca^2+^ influx (Figure 1B), showing a minimal 7% reduction in AUC (Figure S1B). To test the involvement of the GluN2B subtype of NMDAR, we used ifenprodil a non-competitive antagonist for GluN2B (GluN2B IC_50_ = 0.15 µM; GluN2A IC_50_ = 39 µM; GluN2C IC_50_ = 29 µM; GluN2D IC_50_ = 76 µM) [57]. Ifenprodil (3 µM) inhibited NMDA-induced Ca^2+^ influx (Figure 1C), with a 40% reduction in AUC (Figure S1C). Ifendopril is an atypical non-competitive antagonist at GluN1A/GluN2B receptors that similarly had modest effects on the transient peak but reduced the plateau phase of the response to 30 μM NMDA exposure [59]. To test the involvement of GluN2C/2D in NMDA-induced Ca^2+^ influx, we utilized QNZ-46, a non-competitive antagonist for GluN2C and GluN2D (GluN2C IC_50_ = 6 µM; GluN2D IC_50_ = 3 µM; GluN2A IC_50_ = 229 µM; GluN2B IC_50_ > 300 µM) [57]. QNZ-46 (30 µM) did not inhibit the NMDA-induced Ca^2+^ influx (Figure 1D), which showed a 5% reduction in AUC (Figure S1D). Given the absence of either GluN2C or GluN2D subtype-selective antagonists, we next utilized a genetic approach using GluN2C or GluN2D knockout mice with a C57BL/6 background strain as the control. NMDA-induced Ca^2+^ influx was unchanged in both GluN2C and GluN2D knockout mice and was effectively blocked by MK-801 and ifenprodil (Figure 1E–G).

### 2.2. PbTx-2 and NMDA Exposure Produced Distinct Ca^2+^ Influx Pathways

**Figure 2:**
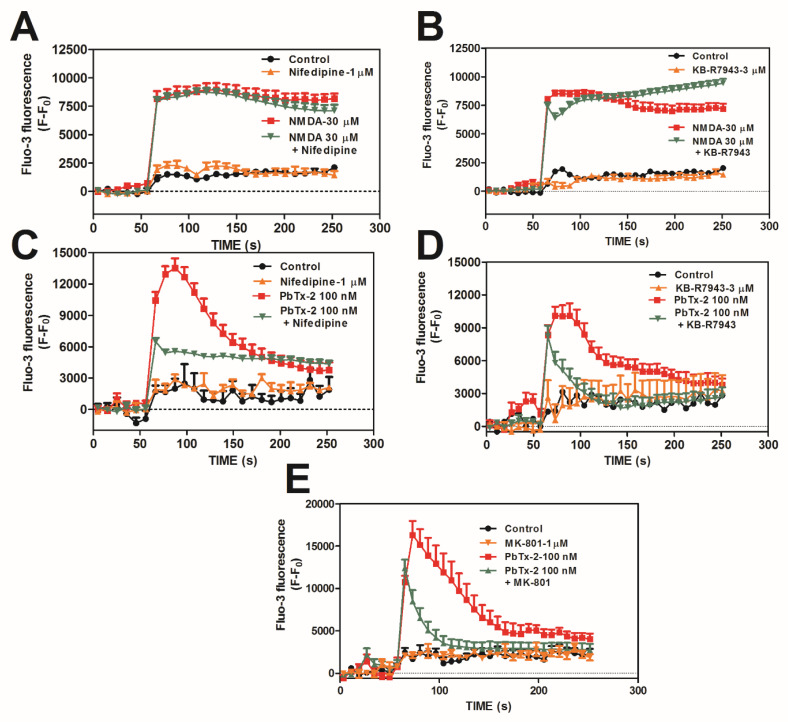
NMDA and PbTx-2 exposure triggers distinct Ca^2+^ influx pathways. NMDA-induced Ca^2+^ influx is mediated exclusively by NMDARs with an absence of an effect by the L-type VGCC inhibitor nifedipine (**A**) or the reverse sodium–calcium exchange inhibitor KB-R7943 (**B**), whereas PbTx-2-induced Ca^2+^ influx involves L-type Ca^2+^ channels, as shown with nifedipine (**C**); sodium Ca^2+^ exchanger, as shown with KB-R7493 (**D**); and NMDARs, as shown with MK-801 (**E**). This experiment was repeated in triplicate in DIV 10–12 cerebrocortical neurons. Data shown represent the mean ± SEM of 3 experiments.

To compare NMDA and PbTx-2-induced Ca^2+^ influxes, we used a pharmacologic approach. Neither 1,4-dihydro-2,6-dimethyl-4-(2-nitrophenyl)-3,5-pyridinedicarboxylic acid dimethyl ester (nifedipine 1 µM; L-type VGCC blocker, Figure 2A) nor 2-[2-[4-(4-nitrobenzyloxy)phenyl]ethyl]isothiourea methane sulfonate (KB-R7943 3 µM; reverse sodium–calcium exchanger inhibitor; NCX_rev_) affected the NMDA-induced Ca^2+^ influx (Figure 2B). Both nifedipine and KB-R7943 had minimal effects on the NMDA-induced Ca^2+^ influx, with nifedipine showing a 6% reduction (Figure S2A) and KB-R7943 displaying a 7% increase (Figure S2B). We next assessed the effect of PbTx-2 alone on Ca^2+^ dynamics in DIV 10–12 cerebrocortical neurons. PbTx-2 (100 nM) produced an increase in [Ca^2+^]_i_, which was partially blocked by nifedipine (Figure 2C), KB-R7943 (Figure 2D), and MK-801 (Figure 2E). All three inhibitors displayed a substantial reduction in the PbTx-2-induced Ca^2+^ influx, with nifedipine showing a 32% reduction (Figure S2C), KB-R7493 showing a 54% reduction (Figure S2D), and MK-801 displaying a 48% reduction. KB-R7943 is an isothiourea derivative that has been shown in cardiac ventricular cells to preferentially inhibit the reversed operation of the Na^+^/Ca^2+^ exchanger (IC_50_ = 0.32 μM) while inhibiting with lower potency voltage-gated sodium channels (IC_50_ = 14 μM). In the present study, KB-R7943 was used at a concentration of 3 μM to minimize its effect on the voltage-gated sodium channel function while maximizing Na^+^/Ca^2+^ exchanger inhibition. At this concentration, KB-R7943 would be expected to produce a greater-than–90% fractional occupancy of the Na^+^/Ca^2+^ exchanger and a less-than–20% fractional occupancy of voltage-gated sodium channels [60,61]. It is, therefore, reasonable to infer that the primary effect of 3 μM KB-R7943 is to inhibit the reversed mode of the Na^+^/Ca^2+^ exchanger function.

### 2.3. PbTx-2-Induced Ca^2+^ Influx Involves the GluN2B but Not GluN2A Subtype of NMDAR

**Figure 3:**
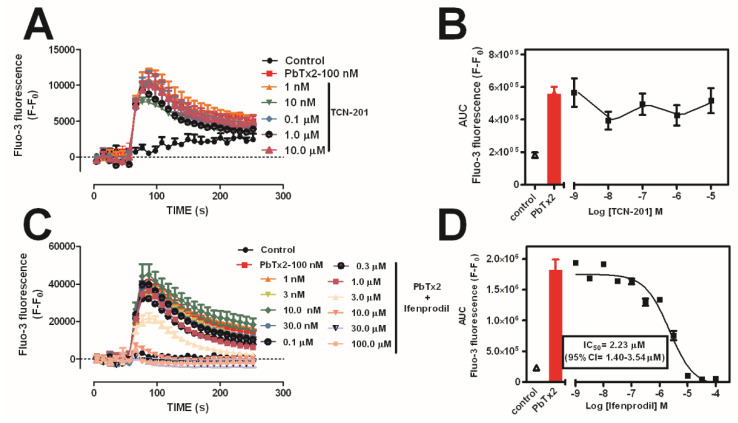
PbTx-2-induced Ca^2+^ influx involves the GluN2B but not GluN2A subtype of NMDAR. PbTx-2-induced Ca^2+^ influx does not involve the GluN2A subtype of NMDARs, as shown by a lack of effect of the non-competitive GluN2A antagonist TC-201 (**A**,**B**). Ifenprodil, a selective GluN2B receptor antagonist, inhibited PbTx-2-induced Ca^2+^ influx in a concentration-dependent manner (**C**,**D**). This experiment was repeated in triplicate in DIV 10–12 cerebrocortical neurons. Data shown represent the mean ± SEM of 3 experiments.

We have previously demonstrated that PbTx-2 is capable of sensitizing cerebrocortical neurons to NMDA-induced Ca^2+^ influx by demonstrating that the NMDA concentration–response curve for Ca^2+^ influx was significantly leftward shifted by 30 nM PbTx-2 [6]. In mature hippocampal and cortical neurons, both GluN2A and GluN2B are expressed and NMDARs consist of a combination of GluN1/GluN2B, GluN1/GluN2A, and GluN1/GluN2A/GluN2B heteromers [62]. Thus, we examined the role of GluN2A and GluN2B subtypes of NMDAR in PbTx-2-induced Ca^2+^ influx. TCN-201 did not block PbTx-2-induced Ca^2+^ influx (Figure 3A,B); however, ifenprodil concentration-dependently blocked the [Ca^2+^]_i_ response to PbTx-2 (Figure 3C,D), with IC_50_ = 2.2 µM (95% CI = 1.4–3.5 µM). Notably, this ifenprodil IC_50_ is in close agreement with the IC_50_ of ifenprodil for the GluN2B subtype of NMDAR.

### 2.4. PbTx-2-Induced Neurite Outgrowth in Cerebrocortical Neurons Is Antagonized by Ifenprodil

**Figure 4:**
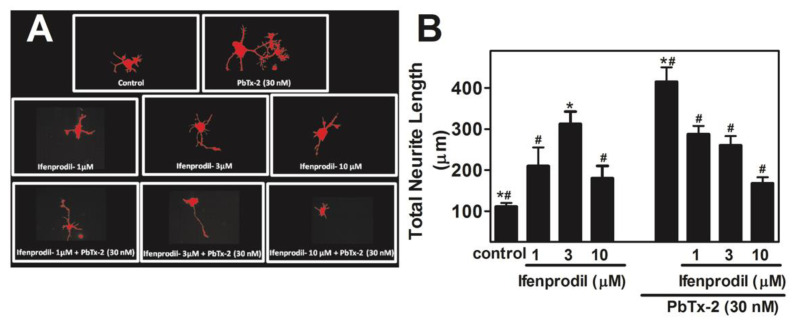
PbTx-2-induced neurite outgrowth in cerebrocortical neurons is antagonized by ifenprodil. Representative images of DIV1 cerebrocortical neurons (**A**). Quantification of neurite length using IMARIS software (**B**). The experiment was repeated twice, and 25 to 30 neurons were quantified for each exposure condition. (* one-way ANOVA, followed by Dunnett’s multiple comparison test, with control *p* < 0.0001; ^#^ one-way ANOVA, followed by Dunnett’s multiple comparison test, with PbTx-2 30 nM *p* < 0.0001). Data shown represent the mean ± SEM of 2 experiments.

We next sought to determine the functional consequences of PbTx-2-induced augmentation of Ca^2+^ influx and NMDAR signaling. Previously, we demonstrated a robust effect of 30 nM PbTx-2 on neurite length [6]. Here, we set out to characterize the involvement of the GluN2B subtype of NMDAR using different concentrations of ifenprodil (1, 3, and 10 µM). PbTx-2 significantly enhanced total neurite outgrowth in immature cerebrocortical neurons with a 30 nM concentration, producing a robust, approximately 4-fold increase in total neurite length (Figure 4A,B). All three ifenprodil concentrations tested significantly reduced the effect of PbTx-2 on neurite outgrowth (control: 111.0 ± 8.9 μm; PbTx-2: 414.7 ± 135.3 μm; PbTx-2 + ifenprodil 1 µM: 287.7 ± 19.7 μm (* *p* < 0.001); PbTx-2 + ifenprodil 3 µM: 260.0 ± 22.3 μm (* *p* < 0.0001); and PbTx-2 + ifenprodil 10 µM: 167.9 ± 14.6 μm (* *p* < 0.0001). As shown in Figure 4B, ifenprodil treatment alone increased neurite outgrowth in a biphasic pattern. A previous report has shown that ifenprodil potentiates nerve-growth-factor-induced neurite outgrowth in PC12 cells [63].

### 2.5. PbTx-2-Induced Neurite Outgrowth in Cerebrocortical Neurons Is Antagonized by a CaMKII Inhibitor

**Figure 5:**
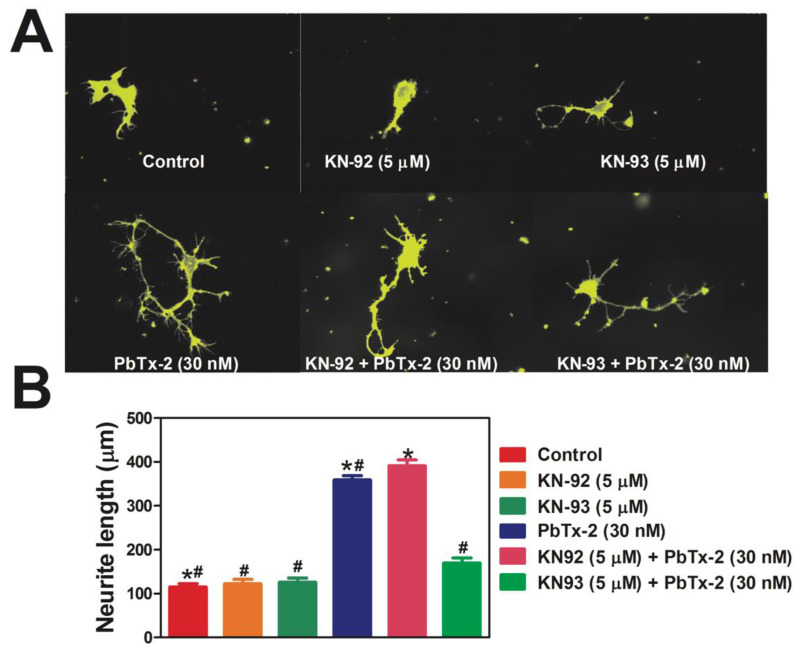
PbTx-2-induced neurite outgrowth in cerebrocortical neurons is antagonized by a CaMKII inhibitor. Representative images of DIV1 cerebrocortical neurons (**A**). Quantification of neurite length using IMARIS software (**B**). The experiment was repeated twice, and 25 to 30 neurons were quantified for each exposure condition. (* one-way ANOVA, followed by Dunnett’s multiple comparison test, with control *p* < 0.0001; ^#^ one-way ANOVA, followed by Dunnett’s multiple comparison test, with PbTx-2 30 nM *p* < 0.0001). Data shown represent the mean ± SEM of 2 experiments.

Previously, we have shown that acute exposure to PbTx-2 (100 nM) produces rapid and sustained phosphorylation of CaMKII (Thr286) [64]. Here, we investigate the involvement of CaMKII in PbTx-2-induced neurite outgrowth. CaMKII can be directly inhibited by KN-93 (IC_50_ = 0.37 µM), which is an antagonist of the calmodulin-binding site in a select group of kinases [65], by binding to Ca^2+^/CaM and indirectly preventing CaMKII activation [66]. KN-92, which is an inactive analog of the CaMKII inhibitor, was used as a negative control [67]. KN-93 treatment significantly reduced the effect of PbTx-2 on neurite outgrowth (Figure 5A,B; control: 115 ± 7 μm; KN-92: 122 ± 10 μm; KN-93: 126 ± 9 μm; PbTx-2: 358 ± 10 μm; PbTx-2 + KN-92, 5 µM: 390 ± 14 μm and PbTx-2 + KN-93, 5 µM: 169 ± 11 μm; * *p* < 0.0001).

### 2.6. PbTx-2-Induced Neurite Outgrowth Involves Rac1 but Not Rho A

**Figure 6:**
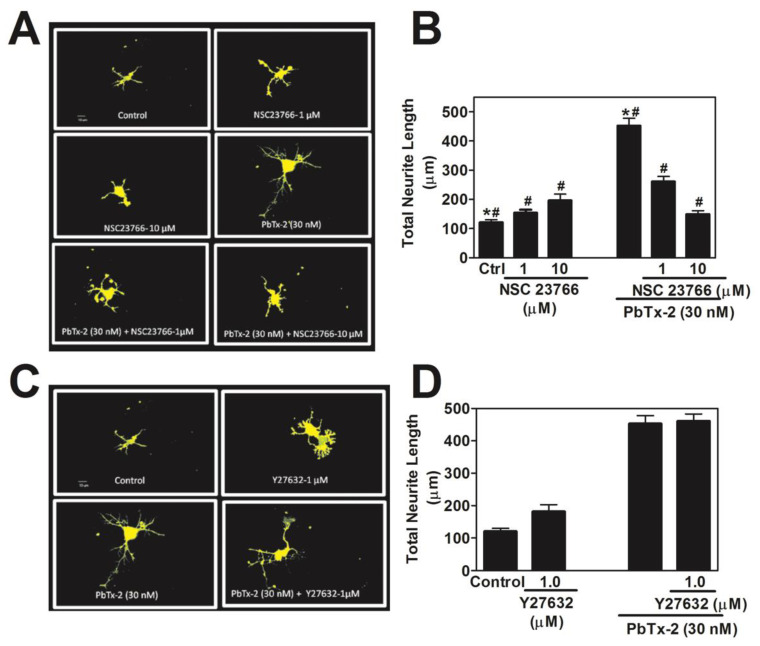
PbTx-2-induced neurite outgrowth involves Rac1 but not Rho A. Representative images of DIV1 cerebrocortical neurons (**A**,**C**). Quantification of neurite length using IMARIS software (**B**,**D**). The experiment was repeated twice, and 25 to 30 neurons were quantified for each exposure condition. (* one-way ANOVA, followed by Dunnett’s multiple comparison test, with control *p* < 0.0001; ^#^ one-way ANOVA, followed by Dunnett’s multiple comparison test, with PbTx-2 30 nM *p* < 0.0001). Data shown represent the mean ± SEM of 2 experiments.

Rho family GTPases play an important role in the regulation of actin dynamics. The members of the Rho GTPase family that are well studied are Cdc42, Rac1, and RhoA. To explore their role in PbTx-2-induced neurite outgrowth, we used a Rac1 selective inhibitor NSC23766 that inhibits Rac1–GEF interaction and, thus, the activation of Rac GTPase. In vitro studies have shown that NSC23766 inhibits the Rac1 binding and activation via Rac-specific GEF Trio or Tiam 1 (IC_50_ ~ 50 μM) without altering RhoA or Cdc42 binding or activation [68]. To block the Rho pathway, we utilized Y27632 (K_i_ = 0.14 µM), a selective inhibitor of the Rho kinase [69]. The Rac1 inhibitor NSC23766 blocked PbTx-2-induced neurite outgrowth (Figure 6A,B), but the Rho inhibitor Y27632 was without an effect (Figure 6C,D).

### 2.7. PbTx-2-Induced Neurite Outgrowth Involves PAK1

**Figure 7:**
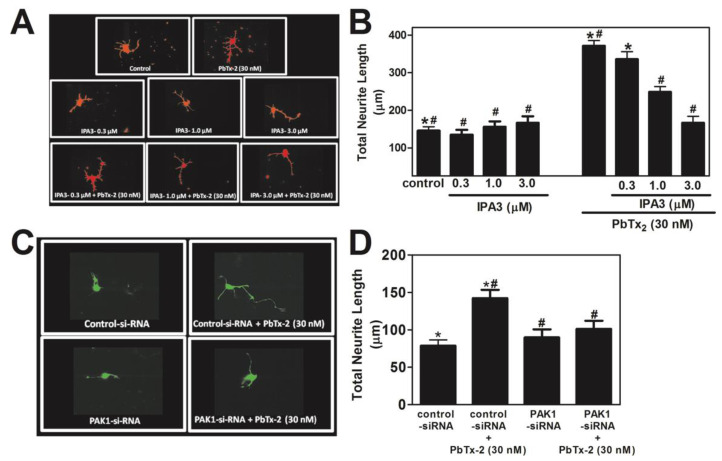
PbTx-2-induced neurite outgrowth involves PAK1. Representative images of DIV1 cerebrocortical neurons (**A**,**C**). Quantification of neurite length using IMARIS software (**B**,**D**). Both experiments were repeated twice, and 25 to 30 neurons were quantified for each exposure condition. (* one-way ANOVA, followed by Dunnett’s multiple comparison test, with control *p* < 0.0001; ^#^ one-way ANOVA, followed by Dunnett’s multiple comparison test, with PbTx-2 30 nM *p* < 0.0001). Data shown represent the mean ± SEM of 2 experiments.

**Figure 8:**
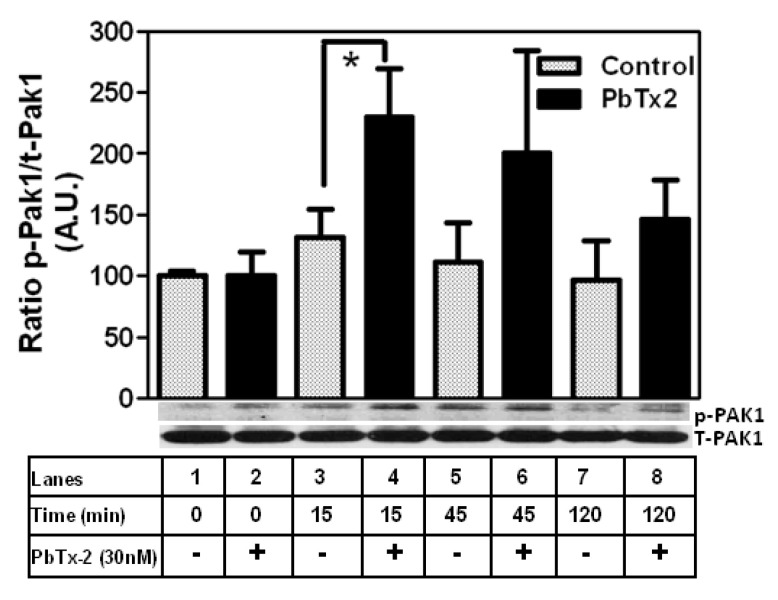
PbTx-2 treatment causes increased phosphorylation of p-PAK1. PbTx-2 treatment leads to increased phosphorylation of PAK T212, which peaks at 15 min. Each bar indicates a quantitative analysis of the relative band densities of immunoblots. Each bar represents mean ± SEM (* *p* < 0.05; *n* = 3).

Active Rac1 is able to regulate actin cytoskeleton assembly by targeting the serine/threonine p21-activated kinase (PAK), a downstream molecule critical in cytoskeleton rearrangement [70,71]. To test the involvement of PAK we treated cerebrocortical cultures with different concentrations of IPA-3 (0.3, 1.0, and 3.0 µM), a pharmacological inhibitor of group I PAKs. IPA-3 is a direct, non-competitive inhibitor of PAK1 [72]. PbTx-2-induced neurite outgrowth was significantly reduced in the presence of IPA-3 (1.0 and 3.0 µM). IPA-3 treatment significantly reduced the effect of PbTx-2 on neurite length (control: 145.1 ± 10 μm; IPA-3 0.3 µM: 135.0 ± 12 μm; IPA-3 1.0 µM: 156.2 ± 14 μm; IPA-3 3.0 µM: 166.8 ± 17 μm; PbTx-2: 371.8 ± 14 μm; PbTx-2 + IPA-3 0.3 µM: 335.7 ± 20 μm; PbTx-2 + IPA-3 1.0 µM: 218.8 ± 14 μm * *p* < 0.0001; PbTx-2 + IPA-3 3.0 µM: 166.8 ± 17 μm * *p* < 0.0001; Figure 7A,B). To confirm the involvement of PAK1 in PbTx-2-induced neurite outgrowth, cerebrocortical neurons were transfected with a PAK1 pSUPER-GFP plasmid that expressed PAK1 siRNA and green fluorescent protein (GFP) [73]. PbTx-2-induced neurite outgrowth was significantly reduced in the presence of the PAK1-specific siRNA but not with a control siRNA (control siRNA: 78.6 ± 7.7 μm; control siRNA+ PbTx-2: 142.2 ± 11.1 μm; PAK1 siRNA alone: 89.6 ± 10.9 μm; PAK1 siRNA + PbTx-2: 101.1 ± 10.8 μm, * *p* < 0.0001; Figure 7C,D). To test whether PbTx-2 treatment results in the phosphorylation of PAK-1 at T212, we treated cerebrocortical cultures with PbTx-2 (30 nM) and followed the temporal activation of PAK1. PbTx-2 treatment led to increased phosphorylation of PAK T212, which peaked at 15 min post-exposure (Figure 8).

### 2.8. PbTx-2 Induces Release of Glutamate into Extracellular Medium

**Figure 9:**
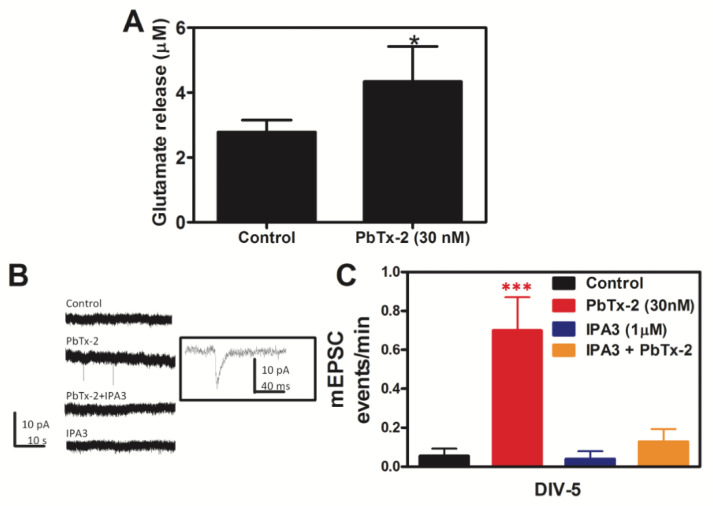
PbTx-2 induces the release of extracellular glutamate. PbTx-2 leads to the elevation of miniature excitatory post-synaptic currents (mEPSCs) and is dependent on PAK1. Pbtx-2 treatment led to an increase in extracellular glutamate release in DIV-5 neurons (* *p* < 0.05) (**A**). Bars in panel A represent the mean ± SD (*n* = 12–30). Representative mEPSC currents in DIV-5 cerebrocortical neurons treated with ± PbTx-2 alone or a Pak1 inhibitor IPA3 (**B**). Histogram summary (mean ± SEM) of mEPSC data for different treatment groups (*** *p* < 0.0003) (**C**). The technical replicates were as follows: control = 9; PbTx-2 = 9; IPA3 = 5; IPA3 + PbTx-2 = 7.

PbTx-2 (30 nM) treatment led to a functional consequence, as demonstrated by the increase in neurite outgrowth. We next examined the release of glutamate into extracellular medium follows PbTx-2 treatment. PbTx-2 (30 nM) was used to provoke glutamate release in murine cerebrocortical neurons. Extracellular glutamate levels were measured by collecting the culture medium of primary neurons that had been exposed to either vehicle or PbTx-2 (30 nM). Glutamate levels were quantified via a glutamate ELISA assay. Our result demonstrated that PbTx-2 (30 nM) caused an approximately two-fold increase in glutamate release compared to the vehicle-treated group (Figure 9A).

### 2.9. PbTx-2-Induced Elevation of Miniature Excitatory Post-Synaptic Current (mEPSCs) Is Dependent on PAK1

Spontaneous glutamate mEPSCs are responses to the release of a single quantum of the excitatory neurotransmitter glutamate. Given the previous demonstration that PbTx-2 exposure enhanced synaptogenesis in immature cerebrocortical neurons, we determined the effect of PbTx-2 on mEPSCs. PbTx-2 increased mEPSCs in DIV-5 cerebrocortical neurons, reflecting accelerated synaptogenesis, and this effect was abrogated by IPA-3 (Figure 9B,C). Based on experimental evidence, we hypothesized that exposure to PbTx-2 increases neurite outgrowth via an NMDA receptor and PAK1 mechanism. The assessment of mEPSC was to address the formation of functional synapses. Recordings of mEPSC at −70 mV would primarily assess AMPA receptor currents, which are observed at mature synapses rather than, for example, silent synapses that may contain NMDA receptors but lack AMPA receptors. PbTx-2 facilitated the formation of functional synapses, as evidenced by the increase in mEPSC events, and IPA3 inhibited this effect, suggesting dependence on PAK1.

### 2.10. PbTx-2 Enhancement of Dendritic Complexity Requires PAK1

**Figure 10:**
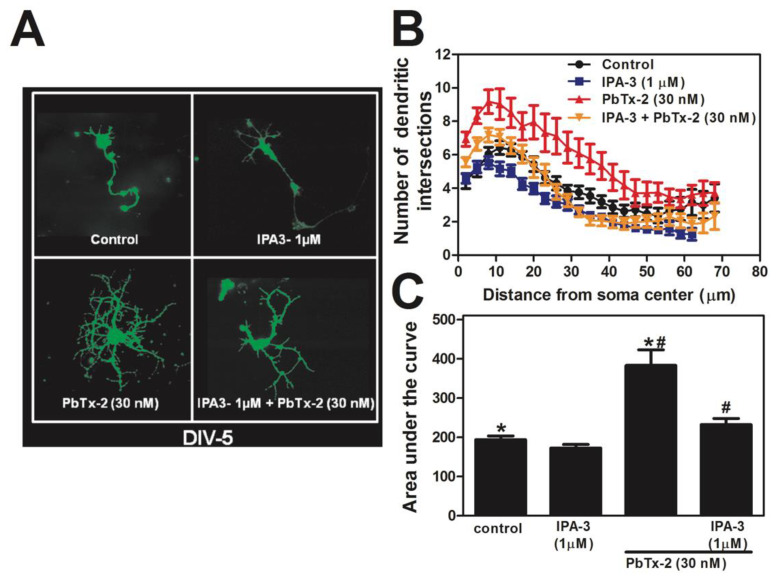
PbTx-2 enhancement of dendritic complexity requires PAK1. Representative images of DIV 5 neurons (**A**). Sholl analysis, performed by IMARIS software, of DIV 5 neurons treated with ± PbTx-2 alone or with the Pak1 inhibitor IPA-3 (**B**). Histogram summary (mean ± SEM) for the area under the curve analysis of Sholl data (**C**). The experiment was performed two times with independent cultures, and 25 to 30 neurons were quantified for each exposure condition. (* one-way ANOVA, followed by Dunnett’s multiple comparison test, with control *p* < 0.05; ^#^ one-way ANOVA, followed by Dunnett’s multiple comparison test, with PbTx-2 30 nM *p* < 0.05).

To test the involvement of PAK1 in PbTx-2-induced increases in dendritic arborization, cerebrocortical neurons were treated with PbTx-2 in the presence and absence of IPA-3. In DIV-5 control neurons, Sholl analysis indicated a gradual increase in branch complexity as a function of distance from the soma, reaching a maximum of 6.5 ± 0.3 (control) and 6.4 ± 0.3 (IPA-3 1 µM) intersections per neuron at 11 µm (Figure 10A,B). PbTx-2 (30 nM) produced a robust increase in dendritic complexity, reaching a maximum of 10.0 ± 0.7 intersections per neuron at 11 µm. IPA-3 pretreatment reduced this effect of PbTx-2 (30 nM) by 7.0 ± 0.4 intersections per neuron at 11 µm. An area under the curve (AUC) analysis of these Sholl data showed a significant increase in dendritic complexity following 30 nM PbTx-2 compared with control (ANOVA, * *p* < 0.05; Dunnett’s post hoc test), which was blocked by IPA-3 (Figure 10C).

### 2.11. PbTx-2 Treatment Produces Phosphorylation of Cofilin and Increases F-Actin Density

**Figure 11:**
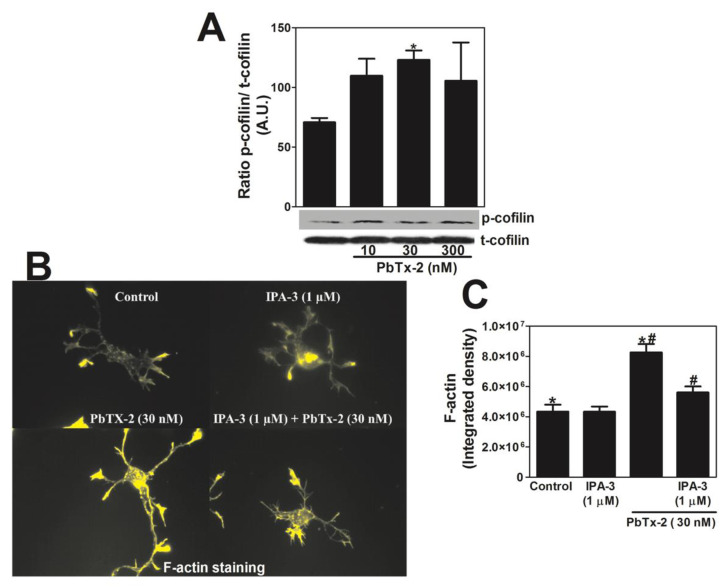
PbTx-2 treatment increased the phosphorylation of cofilin and F-actin density. PbTx-2 causes increased phosphorylation of cofilin in DIV5 cerebrocortical neurons (**A**). Representative images of DIV 1 cerebrocortical neurons (**B**). PbTx-2 treatment leads to an increase in F-actin density, which is blocked by IPA-3 (**B**,**C**). The experiment was performed two times with independent cultures. (* one-way ANOVA, followed by Dunnett’s multiple comparison test, with control *p* < 0.05; ^#^ one-way ANOVA, followed by Dunnett’s multiple comparison test, with PbTx-2 30 nM *p* < 0.05). Data shown represent the mean ± SEM of 2 experiments.

Next, we assessed the effect of PbTx-2 on actin dynamics. Cofilin has been implicated as an important regulator of synaptic plasticity under both physiological and pathological conditions [74,75]. The phosphorylation of cofilin suppresses its activity and has been shown to promote the development of mature spines [76,77]. To explore the role of PbTx-2-induced changes in actin dynamics, DIV-5 cerebrocortical neurons were treated with different concentrations of PbTx-2 (10, 30, and 300 nM). PbTx-2 treatment showed a modest enhancement in the phosphorylation of cofilin in DIV 5 cerebrocortical neurons (Figure 11A). We then assessed the response of F-actin following PbTx-2, IPA-3, or both treatments in DIV-1 cerebrocortical neurons stained with phalloidin and quantified using Image J. PbTx-2 treatment led to an increase in F-actin density, which was blocked by IPA-3 (Figure 11B,C).

## 3. Discussion

Neuronal activity plays a key role in regulating the morphology and connectivity of neurons during development. The mechanisms by which neurons decode this neuronal activity into the activation of signaling pathways that regulate morphological complexity are not fully understood. These pathways are reported to involve local (modulation of actin dynamics) as well as global (activation of nuclear signaling and gene transcription) signaling mechanisms [12]. The elevation of intracellular sodium in dendrites and spines as a consequence of neuronal activity affects NMDAR function and activity-dependent synaptic plasticity [78].

We have previously shown that Ca^2+^ entry in neurons exposed to PbTx-2 occurs through three primary routes: NMDA receptor ion channels, L-type Ca^2+^ channels, and the reversal of the Na^+^/Ca^2+^ exchanger [61]. The NMDA-receptor-dependent increment in [Ca^2+^]_i_ represents a relatively small fraction of the total Ca^2+^ load induced by PbTx-2 exposure. In contrast, when exposed to an NMDA receptor agonist such as L-glutamate, the increase in [Ca^2+^]_i_ appears to be almost entirely through the NMDA receptors [61].

PbTx-2 treatment mimics activity-dependent structural plasticity in that it produces an increase in dendritogenesis, spinogenesis, and synaptogenesis [6,64]. Given the dependence of PbTx-2-induced structural plasticity on NMDAR function, here, we have addressed the NMDAR subtype and downstream signaling mechanisms by which sodium channel activators influence neuronal morphology in DIV 1 and 5 cerebrocortical neurons. Inasmuch as synaptic activity has been shown to elevate [Na^+^]_i_ [79] and sodium acts as a positive regulator of NMDAR function [78], we used the sodium channel activator PbTx-2 as a probe to explore the influence of sodium on NMDAR signaling.

Although NMDARs represent a key source of Ca^2+^ entry following PbTx-2 exposure, the NMDAR subtype responsible for this response was unknown. In this report, we show that PbTx-2-induced Ca^2+^ influx and neurite length were blocked by a GluN2B subtype-selective antagonist in cerebrocortical neurons (Figure 1, Figure 2, Figure 3 and Figure 4). Notably, GluN2B-containing NMDARs have higher surface mobility than GluN2A-containing receptors [80]. We have previously reported that PbTx-2 exposure leads to increased surface expression of GluN2B, suggesting the involvement of this subtype of NMDAR in Ca^2+^ influx [3]. GluN2B receptors have been shown to increase spine and filopodia motility [81,82], which are important for establishing synaptic connections [83]. Moreover, the GluN2B antagonist ifenprodil has been shown to prevent NMDA-induced filopodia formation [84]. A reduction in the number of functional synapses was indicated by a significant reduction in the frequency of mEPSCs and significantly lower spine density [82].

Many of the effects of neuronal activity on synaptic plasticity are mediated by Ca^2+^-dependent signaling events [85]. Ca^2+^/CaM dependent protein kinases (CaMKs) appear to be key mediators of Ca^2+^-dependent neurite outgrowth [86]. Members of the CaMK family are activated in response to GluN receptor stimulation and following neuronal activity [40]. Among the CaMKs, CaMKII is abundantly present in the CNS, with a wide repertoire of substrates, including cytoskeletal proteins. Additionally, GluN2B is reported to be a key activity-dependent recruiter of CaMKII to post-synaptic sites [87]. Such activity-dependent incorporation of CaMKII into post-synaptic sites may play a role in structural and functional synapse maturation during development as well as in learning and memory [88]. Our results (Figure 5) are consistent with previous findings where Ca^2+^/calmodulin-dependent protein kinase II was shown to promote neurite outgrowth in Neuro-2a cells [89,90]. Likewise, inhibition of the CaMK family with KN-93 has been associated with a reduction in synaptic plasticity [65,67].

The Rho family of small GTPases, which includes RhoA, Rac1, and Cdc42, promote morphological changes during neuronal development, including neurite outgrowth, axonal guidance, and dendritic development [91,92]. Rac1 activation is closely coupled to the activation of Cdc42 [93], allowing for the coincident and coordinated formation of filopodia and lamellopodia. NMDAR stimulation is able to regulate Rac1-dependent actin remodeling, which is important for the development and structural remodeling of dendritic arbors and spines [94,95]. Here, we show that PbTx-2 feeds into the Rac1 pathway to induce neurite outgrowth and that blocking RhoA does not inhibit PbTx-2-induced neurite outgrowth (Figure 6). Our results are consistent with previous findings, where it has been generally demonstrated that RhoA inhibits, whereas Rac1 and Cdc42 promote, the growth and stability of dendritic spines [96]. One study using Rac1 inhibitor NSC23766 in rat cortical neurons showed this inhibitor might function as an NMDAR antagonist instead of inhibiting cytosolic Rac1; however, in that study, the authors used a considerably higher concentration of NSC 23766 (100 µM) [97], whereas we used 1 and 10 µM NSC23766 concentrations. Using higher concentrations of this inhibitor might contribute to off-target effects of this Rac1 antagonist.

PAK1 is a key downstream effector of Rac1 [70]. PAK1 was originally identified in a Rho-GTPase screen, where it was complexed with activated GTP-Rac1 [70]. PAK1 is a serine/threonine kinase involved in cellular activities such as cytoskeletal dynamics, cell migration, neurogenesis, angiogenesis, mitosis, apoptosis, and transformation [98,99,100,101,102,103]. PAK kinases are effectors of Rac1 and have been shown to play an important role in neurite initiation and outgrowth [104]. Inhibition of PAK1 leads to a decrease in basal NMDAR currents [105]. Our result suggests that PbTx-2-induced neurite outgrowth involves PAK1 since inhibition by both a non-competitive inhibitor of PAK1 (IPA-3) and PAK1 siRNA blocks PbTx-2-induced neurite outgrowth (Figure 7). Further, PAK1 has a phosphorylation site on T212, a site absent in PAK2 and PAK3 [106]. Areas of embryonic mouse brain demonstrate differential expression of phosphorylated PAK-T212, which is present primarily in the cerebral cortex, hippocampus, and thalamus. Increased PAK1-T212 phosphorylation plays a specific role in neurons when they are undergoing extensive cytoskeletal changes, such as during axonal outgrowth [107]. Furthermore, it has been demonstrated that mice treated with the Rac1 inhibitor showed a substantial reduction of PAK1-T212 phosphorylation levels, providing direct evidence that Rac1 regulates PAK1-T212 phosphorylation [108]. We found that PbTx-2 treatment increases the phosphorylation of PAK1 at T212, which implicates a role for this phosphorylation site in PbTx-2-induced neurite outgrowth (Figure 8). Future studies are warranted to explore additional phosphorylation sites on PAK1 that may be involved in response to PbTx-2 exposure.

Dissociated neurons in culture form a network of synaptically connected cells, and spontaneous Ca^2+^ oscillations that are thought to be due to the rhythmic release of glutamate. Such synchronized Ca^2+^ oscillations in primary neuronal cultures measured at the population level with Ca^2+^-sensitive fluorescent probes have been strictly associated with bursts of action potentials [109]. Previously, we reported that PbTx-2 exposure in cerebrocortical neurons produces an increase in [Na^+^]_i_, an up-regulation of NMDAR function, acceleration of the emergence of spontaneous Ca^2+^ oscillations, and the engagement of downstream Ca^2+^ dependent signaling pathways [64]. Our results herein demonstrate that PbTx-2 treatment provoked the release of glutamate (Figure 9A), and this increment in extracellular glutamate was sufficient to cause an increase in spontaneous Ca^2+^ oscillations and neurite outgrowth in cerebrocortical neurons.

One of the hallmarks of synaptic scaling is the frequency of mEPSCs [110]. Previously, we reported that PbTx-2 treatment increased synaptic density [64]. In this report, PbTx-2 treatment led to an increase in mEPSCs events, which were blocked by IPA-3, confirming that PbTx-2 treatment caused increased synapse formation with the involvement of PAK1 (Figure 9B,C). We have also shown that PbTx-2 30 nM treatment increased dendritic arborization and filopodia formation [64]. Our results suggest that PbTx-2-induced neurite outgrowth involves PAK1 (Figure 10), inasmuch as IPA-3 blocks dendritic arborization.

A major downstream effector of PAK1 is cofilin, an actin-binding protein essential for controlling the equilibrium between filamentous and monomeric actin [111]. Cofilin is inactivated by LIM-kinase-mediated phosphorylation (LIM kinase is a substrate of PAK) and is reactivated by cofilin phosphatase [112]. The dephosphorylated cofilin binds to F-actin, leading to actin-severing depolymerization [75,113]. Since the Ser3 residue of cofilin acts as a switch for actin assembly (F-actin stabilization) and disassembly (F-actin severing) [114], we used a Ser3-phosphorylated antibody to probe for PbTx-2-induced p-cofilin levels. We found that PbTx-2 causes a significant increase in p-cofilin expression in DIV-5 neurons (Figure 11A) and increased density of F-actin throughout the dendritic arbor and soma (Figure 11B,C) in DIV-1 neurons.

**Figure 12:**
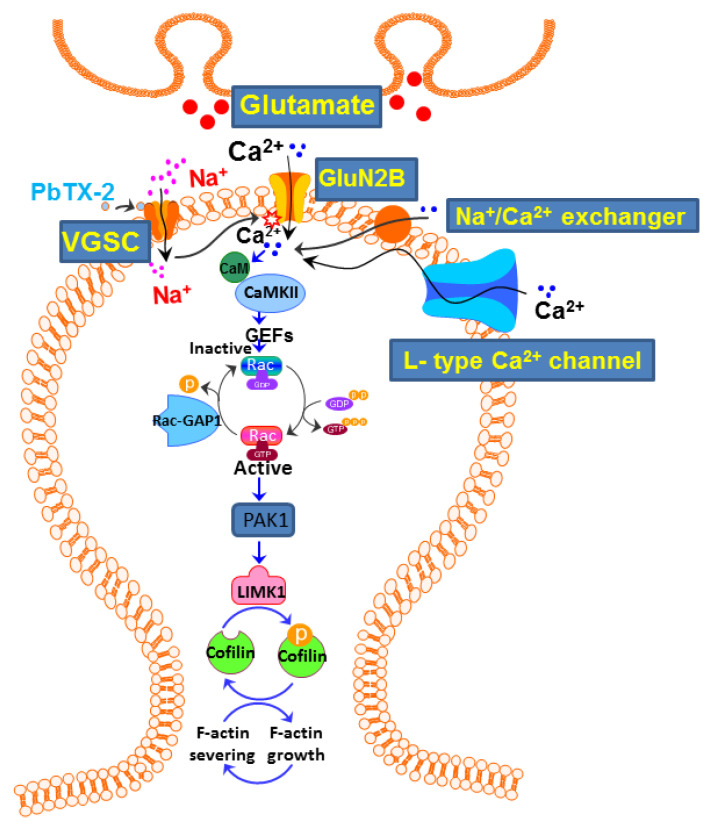
Schematic diagram of the pathways involved in PbTx-2-induced neurite outgrowth. PbTx-2 actions cause increased spontaneous Ca^2+^ oscillations, which lead to the presynaptic release of glutamate. This glutamate release produces an increase in Ca^2+^ influx through NMDARs, leading to the activation of CaMKII and the small G-protein Rac, which, in turn, activates its downstream effector PAK1. The activated Pak1 engages the cofilin cycle, resulting in changes in F-actin filaments, with attendant increases in neurite outgrowth. Abbreviations: VGSC: voltage-gated sodium channels, GluN2B: N-methyl-D-aspartate receptor subtype 2B, CaMKII: Ca^2+^/calmodulin-dependent kinase II; GEF: guanine-nucleotide-exchange factor; PAK1: p21 protein (Cdc42/Rac)-activated kinase1.

Based on these data, we proposed a model for PbTx-2-induced neurite outgrowth (Figure 12) that involves GluN2B–NMDARs. The involvement of this NMDAR subtype leads to the activation of CaMKII, Rac1, and its effector PAK1, causing changes in cofilin activity and the stabilization of F-actin, resulting in the stabilization of the actin cytoskeleton. Thus, VGSC activators may represent a novel pharmacological strategy to promote neuronal plasticity through the NMDAR–GluN2B–CaMKII–Rac1–PAK1-dependent pathway. Together, these data may contribute to the neural repair mechanisms occurring in the PbTx-2-treated post-ischemic stroke mouse model that corresponds with improvements in motor function [7], suggesting a novel pharmacological strategy in the treatment of neurodegenerative and neurological disorders.

## 4. Materials and Methods

### 4.1. Reagents

Trypsin, penicillin, streptomycin, heat-inactivated fetal bovine serum, horse serum, and soybean trypsin inhibitor were obtained from Atlanta Biologicals (Norcross, GA, USA). Minimum essential medium (MEM), deoxyribonuclease (DNase), poly-L-lysine, poly-D-lysine hydrobromide, cytosine arabinoside, NMDA, protease inhibitor cocktail, MK-801, and ifenprodil were purchased from Sigma (St. Louis, MO, USA). Pluronic acid and Fluo-3 AM were purchased from Molecular Probes (Eugene, OR, USA). TCN-201, IPA-3, and NSC23766 were purchased from Tocris (Bristol, UK). Pierce ECL kits (Thermo Fisher Scientific, Rockford, IL, USA), Neurobasal, and B-27 supplements were purchased from Invitrogen Corporation (Carlsbad, CA, USA); the p-PAK1 antibody (Thr 212) from Santa Cruz Biotechnology (Dallas, TX, USA); and the PAK1 antibody anti-rabbit IgG HRP-linked antibody from Cell Signaling Technology (Danvers, MA, USA). Brevetoxin-2 (PbTx-2) was isolated and purified from *Karinia breve* cultures at the Center for Marine Sciences at the University of North Carolina (Wilmington, NC, USA). QNZ-46 was a gift from SF Traynelis, Department of Pharmacology, Emory University, Atlanta, GA. The GluN2D subtype of NMDA receptor knockout mice was obtained from Daniel T. Monaghan, Department of Pharmacology and Experimental Neuroscience, University of Nebraska Medical Center, Omaha, Nebraska.

### 4.2. Cerebrocortical Neuron Culture

Primary cultures of cerebrocortical neurons were harvested from embryos of Swiss-Webster mice on embryonic day 16 and cultured as described previously [2]. Cells were plated onto poly-l-lysine-coated (Sigma-Aldrich, St. Louis, MO, USA) 96-well clear-bottomed, black-well culture plates (MidSci, St. Louis, MO, USA) at a density of 1.5 × 10^5^ cells/mL (150 μL/well), 24-well (15.6 mm) culture plates at a density of 0.05 × 10^6^ cells/mL (0.5 mL/well), or 6-well (35 mm) culture dishes at a density of 2.25 × 10^6^ cells/mL (2 mL/well), respectively, and incubated at 37 °C and 95% humid atmosphere in 5% CO_2_. Cytosine arabinoside (10 μM) was added to the culture medium on day 1 after plating to prevent the proliferation of non-neuronal cells. The culture media was changed on days 4 and 7 using a serum-free growth medium containing Neurobasal medium supplemented with B-27, 100 IU/mL penicillin, 0.1 mg/mL streptomycin, and 0.2 mM L-glutamine. All animal-use protocols were approved by the Creighton University Institutional Animal Care and Use Committee (IACUC).

### 4.3. Intracellular Ca ^2+^ Monitoring

Cerebrocortical neurons grown in 96-well plates were used for intracellular Ca^2+^ concentration ([Ca^2+^]_i_) measurements as previously described [6]. Briefly, the growth medium was removed and replaced with dye loading medium (100 μL per well) containing 4 μM Fluo-3 AM and 0.04% pluronic acid in Locke’s buffer. After 1 h of incubation in the dye loading medium, the neurons were washed four times in fresh Locke’s buffer (150 μL per well, 22 °C) using an automated microplate washer (Bio-Tek Instruments Inc., Winooski, VT, USA) and transferred to a FLEX Station™ II (Molecular Devices, Sunnyvale, CA, USA) benchtop scanning fluorometer chamber. Fluorescence measurements were carried out at 37 °C. The neurons were excited at 488 nm, and a Ca^2+^-bound Fluo-3 emission was recorded at 538 nm at 1.2 s intervals. After recording baseline fluorescence for 60 s, 50 μL of a 4X concentration of NMDA and PbTx-2 or 50 μL of a mixture of antagonist and agonist was added to the cells at a rate of 26 μL/s, yielding a final volume of 200 μL/well; the fluorescence was monitored for an additional 140–240 s. The Fluo-3 fluorescence was expressed as (F_max_−F_0_), where F_max_ is the maximum and F_0_ the baseline fluorescence measured in each well.

### 4.4. Determination of Total Neurite Length and Diolistic Labeling

Cells were plated on poly-lysine-coated 12 mm glass coverslips (Thermo Fisher Scientific, Waltham, MA, USA) and placed inside 24-well culture plates at a low density of 0.05 × 10^6^ cells/mL (0.5 mL/well). To assess the influence of PbTx-2 on neuritogenesis, primary cultures of immature cerebrocortical neurons were exposed to 30 nM PbTx-2 for 24 h, beginning 3 h after plating, and total neurite outgrowth was measured. In some experiments, 30 nM PbTx-2 were co-incubated with ifenprodil, NSC 23766, Y27632, and IPA-3 (Sigma-Aldrich, St. Louis, MO, USA). At 24 h after plating, cultures were fixed at room temperature for 15 min using 1.8% paraformaldehyde in phosphate-buffered saline (PBS). After fixation, neurons were diolistically labeled with DiI [115]. The dye particles were allowed to spread across the neuronal membrane overnight, and coverslips were then mounted for imaging on an Olympus IX 71 inverted microscope with a Himamatsu camera. Digital images of individual neurons were captured, and total neurite length was quantified [64]. To reduce the effect of paracrine neurotrophic factors on neurite growth, only those neurons that were separated from surrounding cells by approximately 150 μm were digitally acquired and analyzed. Digital images of individual neurons were captured and exported as 16-bit images. All neurites in a single neuron, including those from secondary branches, were semi-automatically traced, and the length was measured by using the using Filament Tracer module of IMARIS 6.4.0 software (Bitplane, South Windsor, CT, USA). At least 25–30 randomly chosen neurons from two different cultures were evaluated for each treatment group.

### 4.5. Plasmids and Nucleofection

The PAK1 pSUPER-GFP plasmid and mutated control PAK1 pSUPER-GFP plasmid [73] were generous gifts from Dianqing Wu (University of Connecticut, Farmington). The primary cultures of immature cerebrocortical neurons were transfected with Lipofectamine 2000 (Life Technologies, Grand Island, NY, USA). Dissociated cortical neurons obtained from E16 pups were plated at a density of 0.5 × 10^6^ neurons/well. Two hours post-plating, cells were transfected with 1.0 µg of plasmids containing the gene of interest or mutated control. Three hours post-transfection, cells were treated with 30 nM PbTx-2 or vehicle control. In order to give more time for the expression of genes of interest and to access the influence of PbTx-2 on neuritogenesis, DIV-2 neurons were imaged in experiments involving transfection.

### 4.6. Measurement of Dendritic Complexity

Neurons grown on poly-D-lysine-coated glass coverslips placed inside 24-well culture plates (0.1 × 10^6^ cells per well) were used. To assess the influence of PbTx-2 and PAK1 on dendritic complexity, primary cultures of immature cerebrocortical neurons were exposed to either 30 nM PbTx-2, 1 µM IPA-3, or both for 5 days, beginning 3 h after plating. At 5 days after plating, cultures were fixed at room temperature for 15 min using 1.8% paraformaldehyde in phosphate-buffered saline (PBS). After fixation, neurons were diolistically labeled with DiI [115]. Z-stacked images were acquired using an Olympus spinning-disk confocal microscope, and each neuron was scanned at 0.2 μm intervals along the z-axis with a depth of 5 μm (25 planes). For quantitative analysis, a 3D perspective was rendered by the surpass module of IMARIS software (Bitplane, South Windsor, CT, USA). For the analysis of dendritic arbor complexity, the dendritic tracings were quantified by an automated 3D Sholl analysis [64].

### 4.7. Western Blot

Western blot analysis was performed by using cells grown in six-well plates. For acute experiments, DIV-1 cells were exposed to 30 nM PbTx-2 for 30 min at 37 °C. For pharmacological experiments, cultures were pre-incubated either in the presence or absence of specific antagonists or vehicle for 15 min. At the end of the time period, cultures were transferred onto ice slurry to terminate drug exposure and washed three times with ice-cold PBS. Cells were lysed using ice-cold lysis buffer (50 mM Tris, 50 mM NaCl, 2 mM EDTA, 2 mM EGTA, 1% Nonidet P40, 0.1% SDS, 2.5 mM sodium pyrophosphate, and 1 mM sodium orthovanadate). Phenylmethylsulfonyl fluoride (1 mM) and 1× protease inhibitor mixture (Sigma-Aldrich, St. Louis, MO, USA) were then added, and the lysate was incubated for 30 min at 4 °C. Cell lysates were sonicated and then centrifuged at 13,000× *g* for 15 min at 4 °C. The supernatant was assayed by the Bradford method [116] to determine protein content. Equal amounts of protein were mixed with the Laemmli sample buffer and heated for 5 min at 75 °C. The samples were loaded onto a 10% SDS-polyacrylamide gel electrophoresis gel, transferred to a PVDF membrane, and immuoblotted with specific antibodies. Blots were developed with a Pierce ECL kit (Thermo Fisher Scientific, Rockford, IL, USA) for 2 min. Blots were subsequently stripped (63 mM Tris base, 70 mM SDS, 0.0007% 2-mercaptoethanol, pH 6.8) and reprobed for further use. Western blot densitometry data were obtained using Image J software (NIH, http://imagej.nih.gov/ij/).

### 4.8. Glutamate Release Assay

For the in vitro glutamate release assay, cerebrocortical neurons were grown for 5 days in 6-well plates as described previously [64]. After 5 days in culture, the cells were washed (2×) in Locke’s buffer and incubated in Locke’s for 30 min. Cells were then treated with either vehicle or PbTx-2 (30 nM) for an additional 30 min. Supernatant was collected and immediately processed for glutamate. The glutamate concentration was determined by an enzyme-linked immunosorbent assay (ELISA; Labor Diagnostika Nord, Nordhorn, Germany) in accordance with the manufacturer’s instructions. Samples were analyzed in duplicate on a plate reader (Bio-Tek, Winooski, VT, USA) by measuring the absorbance at 450 nm.

### 4.9. mEPSCs Recording

Whole-cell voltage-clamp recordings were obtained at a holding potential of −70 mV (not corrected for junction potential) from cultured neurons at room temperature (22–25 °C) with an Axopatch 200B (Molecular Devices, Sunnyvale, CA, USA). The osmolarity of the intracellular solution ranged from 280–290 mOsm. Recordings were performed in extracellular Locke’s buffer containing (in mM) 150 NaCl, 3 KCl, 10 HEPES, 6 mannitol, 1.5 MgCl_2_, and 2.5 CaCl_2_ (pH 7.4), supplemented with 100 µM picrotoxin and 1 µM tetrodotoxin to select for miniature excitatory post-synaptic currents (mEPSCs). Recordings were obtained using a glass pipette with a resistance of 5–8 mOhm, filled with a solution consisting of (in mM) 110 cesium gluconate, 30 CsCl_2_, 5 HEPES, 4 NaCl, 0.5 CaCl_2_, 2 MgCl_2_, 5 BAPTA, 2 Na_2_ATP, 0.3 Na_2_GTP, and 5 QX314 (pH 7.35). Whole-cell recordings with a pipette access resistance of less than 20 mOhm and that changed less than 20% during the recording were included. Signals were filtered at 2 kHz and digitized at 10 kHz using an Axon Digidata 1440A analog-to-digital board (Molecular Devices, Sunnyvale, CA, USA). Analysis of mEPSCs was performed using Minianalysis with an amplitude threshold of 5 pA.

### 4.10. F-Actin Staining

Cells were plated on poly-lysine-coated 12- mm glass coverslips (Thermo Fisher Scientific, Waltham, MA, USA) and placed inside 24-well culture plates at a low density of 0.05 × 10^6^ cells/mL (0.5 mL/well). To assess the influence of PbTx-2 and PAK1 on cytoskeleton changes, primary cultures of immature cerebrocortical neurons were exposed to either 30 nM PbTx-2, 1 µM IPA-3, or both for 24 h, beginning 3 h after plating, and F-actin visualization was performed using an F-actin visualization kit (Cytoskeleton Inc., Denver, CO, USA). The DIV-1 cultured cortical neurons were fixed, and F-actin was labeled strictly following the manufacturer’s protocol. Imaging was performed on an Olympus IX 71 inverted microscope with a Himamatsu camera. The density of individual neurons was quantified using Image J software (NIH, http://imagej.nih.gov/ij/).

### 4.11. Statistical Analyses and Graphical Illustration

Data were analyzed and graphical illustrations were generated using GraphPad Prism (La Jolla, CA, USA). Calcium influx analyses were plotted as fluorescence reads over time. The AUC was plotted, and a one-way ANOVA with Dunnet’s multiple comparisons was used to determine statistical significance. Neurite outgrowth was quantified using IMARIS software and then plotted in GraphPad Prism. A one-way ANOVA with Dunnet’s multiple comparisons was used to determine statistical significance, with comparisons to both the positive and negative controls denoted. For mESPCs, a histogram summary was used, showing the mean ± SEM.

## Data Availability

Not applicable.

## Supplementary Materials

The following supporting information can be downloaded at: https://www.mdpi.com/article/10.3390/md20090559/s1. Figure S1: Area under the curve (AUC) was assessed and a one-way ANOVA with multiple comparisons were performed to determine statistical significance for the effects of pretreatment with MK-801, TC-201, ifenprodil, and QNZ-46 on NMDA-induced Ca^2+^ influx. Figure S2: AUC was assessed and a one-way ANOVA with multiple comparisons were performed to determine statistical significance for the effects of pretreatment with nifedipine and KB-R7943 on NMDA-induced Ca^2+^ influx, and nifedipine, KB-R7943, and MK-801 on PbTx-2-induced Ca^2+^ influx. Figure S3. Raw data for p-PAK Western Blot. Figure S4. Raw data for p-cofilin Western Blot.
