# Supplementary material for: Stimulation of Neurite Outgrowth in Cerebrocortical Neurons by Sodium Channel Activator Brevetoxin-2 Requires Both N-Methyl-D-aspartate Receptor 2B (GluN2B) and p21 Protein (Cdc42/Rac)-Activated Kinase 1 (PAK1)
Source: Mar Drugs. 2022 Aug 31;20(9):559. doi: 10.3390/md20090559 (PMC9504648; doi:10.3390/md20090559)
Supplement: Supplementary file 1 [file marinedrugs-20-00559-s001.zip › marinedrugs-1813769-supplementary.pdf]

marine drugs

MDPI

Article

Stimulation of Neurite Outgrowth in Cerebrocortical Neurons by Sodium Channel Activator Brevetoxin-2 Requires Both N-Methyl-D-aspartate Receptor 2B (GluN2B) and p21 Protein (Cdc42/Rac)-Activated Kinase 1 (PAK1)

Suneet Mehrotra [1,2], Marsha L. Pierce [1,3], Shashank M. Dravid [1] and Thomas F. Murray [1,*]

1 Department of Pharmacology and Neuroscience, School of Medicine, Creighton University, Omaha, NE 68178, USA
2 Omeros, Seattle, WA 98119, USA
3 Department of Pharmacology, College of Graduate Studies, Midwestern University, Downers Grove, IL 60515, USA
* Correspondence: tfmurray@creighton.edu; Tel.: +1-402-280-2319

Supplementary Figure S1

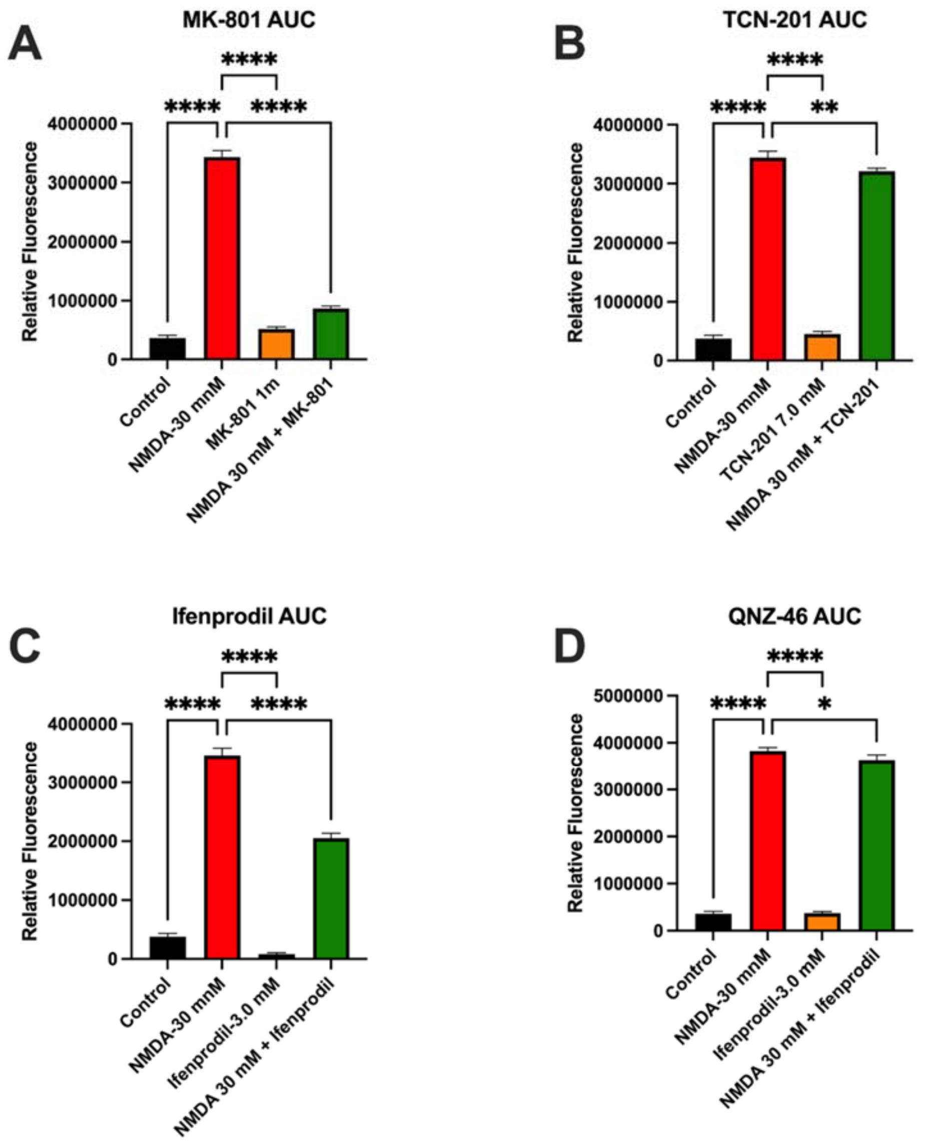

Figure S1. Area under the curve (AUC) was assessed and a one-way ANOVA with multiple comparisons were performed to determine statistical significance for the effects of pretreatment with NMDA-channel blocker MK-801 (**A**), noncompetitive GluN2A antagonist TC-201 (**B**), noncompetitive GluN2B antagonist ifenprodil (**C**), and GluN2C and 2D antagonist QNZ-46 (**D**) on NMDA-induced Ca^{2+} influx (Figure 1A-D). Data shown represent the mean ± SEM of 3 experiments.

Supplementary Figure S2

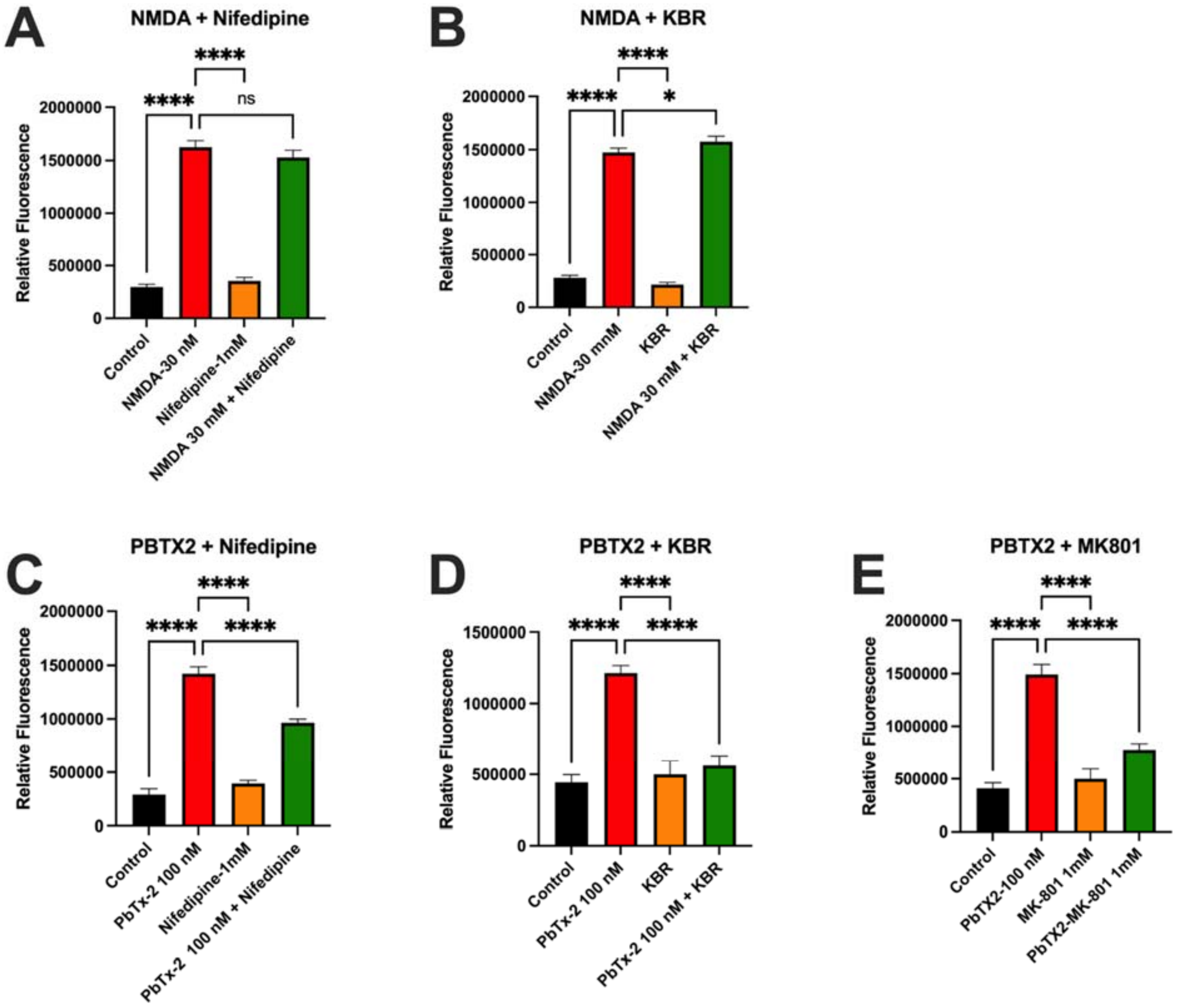

Figure S2. AUC was assessed and a one-way ANOVA with multiple comparisons were performed to determine statistical significance for the effects of pretreatment with nifedipine (**A**), reverse sodium-calcium exchanger KB-R7943 (**B**) on NMDA-induced Ca^{2+} influx and nifedipine (**C**), KB-R7943 (**D**) and MK-801 (**E**) on PbTx-2-induced Ca^{2+} influx (Figure 2A–E). Data shown represent the mean ± SEM of 3 experiments.

Supplementary Figure S3.

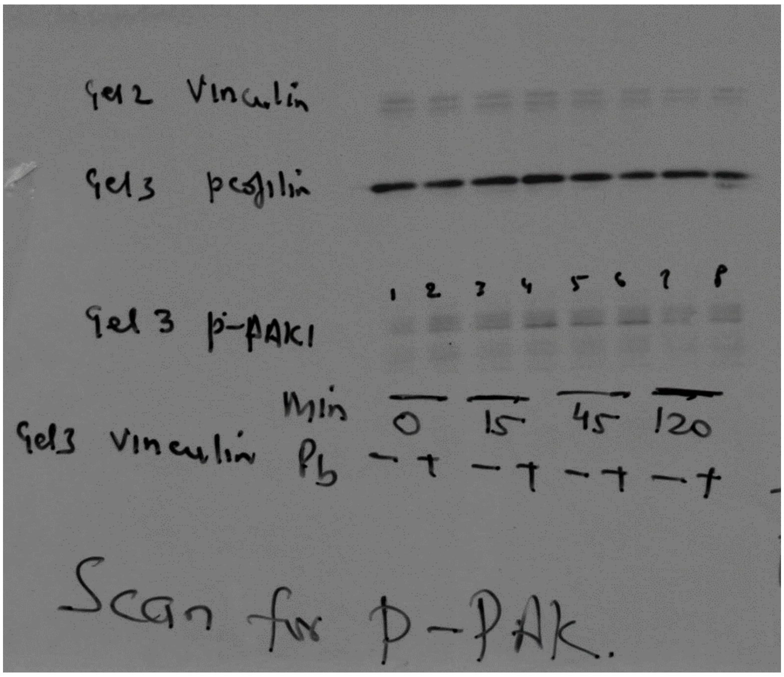

Figure S3. Raw data for p-PAK Western Blot.

Supplementary Figure S4

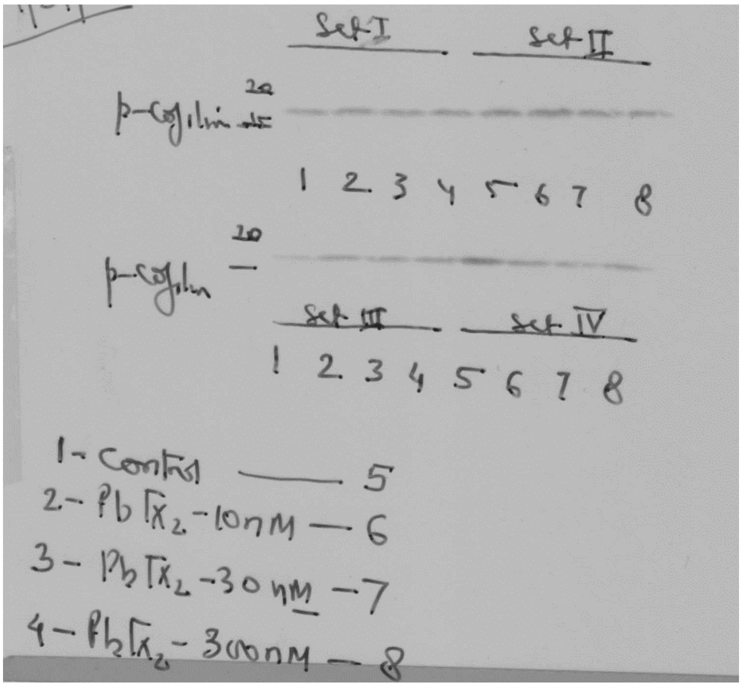

Figure S4. Raw data for p-cofilin Western Blot.
